# Microfabrication and Applications of Opto-Microfluidic Sensors

**DOI:** 10.3390/s110505360

**Published:** 2011-05-18

**Authors:** Daiying Zhang, Liqiu Men, Qiying Chen

**Affiliations:** 1 Department of Physics and Physical Oceanography, Memorial University of Newfoundland, St. John’s, Newfoundland, A1B 3X7, Canada; E-Mail: dz4638@mun.ca; 2 CREAIT Network, Memorial University of Newfoundland, St. John’s, Newfoundland, A1C 5S7, Canada; E-Mail: lmen@mun.ca; 3 Faculty of Engineering and Applied Science, Memorial University of Newfoundland, St. John’s, Newfoundland, A1B 3X5, Canada

**Keywords:** opto-microfluidics, microfluidics, optofluidics, ultrafast laser microfabrication

## Abstract

A review of research activities on opto-microfluidic sensors carried out by the research groups in Canada is presented. After a brief introduction of this exciting research field, detailed discussion is focused on different techniques for the fabrication of opto-microfluidic sensors, and various applications of these devices for bioanalysis, chemical detection, and optical measurement. Our current research on femtosecond laser microfabrication of optofluidic devices is introduced and some experimental results are elaborated. The research on opto-microfluidics provides highly sensitive opto-microfluidic sensors for practical applications with significant advantages of portability, efficiency, sensitivity, versatility, and low cost.

## 1. Introduction

In recent years, increasing effort around the world has been devoted to human health with the development of various novel micro- and nano-technologies, in which the analysis of complex biological systems such as living cells with opto-microfluidic technologies receives significant attention. Meanwhile, opto-microfluidic sensors are gaining increasing acceptance in clinical medicine for bioanalysis and disease diagnosis with continuous emergence of new applications. We use the term “opto-microfluidics” to refer to the research that takes advantages of both optics/photonics and microfluidics. It is obvious that there is no distinct boundary between microfluidics and optofluidics as many techniques and applications are commonly found in these two disciplines. Important topics in the field of opto-microfluidics include: fabrication of micro- and nano- systems for chemical analysis, manipulation of fluids on microchips, integration of microsensors (chemical, biological, optical, photonic, *etc.*) into opto-microfluidic systems, design and modelling of opto-microfluidic devices and systems, and applications of opto-microfluidic systems. In order to reveal new applications of various versatile systems, an overview on the development in this exciting field and the state-of-the-art opto-microfluidic devices and systems will be necessary, which will be of interest to researchers in academia and industry.

The first microfluidic device was a miniaturized gas chromatography (GC) system developed at Stanford University in the 1970s [1]. In the 1980s, the growth of molecular biology such as genomes, DNA, and proteins, stimulated the development of highly sensitive microanalysis devices. A series of microfluidic devices integrated with different assay operations (for example, sample pretreatment and detection) were developed. The so-called Micro Total Analysis System (*μ* TAS) or Lab-on-Chip (LOC) has been the most rapidly growing area due to its unique advantages. Meanwhile, various kinds of microfluidic sensor chips have been reported with different functionalities for different applications. The microfluidic study, which bases on a fluidic platform, possesses many advantages owing to the miniaturization of the devices. These merits include reduced consumption of reagents and analytes, improved time efficiency in the analysis, shrinkage in the size and weight of the systems, increased portability, reduced amount of harmful by-products, and potentially low cost in fabrication. Consequently, microfluidic systems have found a wide range of applications in areas such as molecular analysis, biodefense, molecular biology, microelectronics, clinical diagnostics, and drug development [2]. A few monographs provide good reviews on the history and development in the field of microfluidics [3,4].

During the development of microfluidics, optical components, such as light sources, mirrors, gratings, lenses, and waveguides, have been added into the microfluidic platforms to increase the sensitivity and resolution of the measurement. Starting from years prior to the emergence of microfluidics, particularly in recent years, researchers are exploring various optical techniques for biochemical sensing, such as photonic microresonators [5] and Mach-Zehnder interferometers [6], fiber grating resonators [7], or microstructured fiber sensors [8] in addition to well-known methods such as absorbance, fluorescence, refraction, and Raman-scattering. The term “optofluidics” was coined and gradually adopted by the research community. In 2003, the term “optofluidics” was included in the name of a university research center, *i.e.*, Center for Optofluidic Integration at California Institute of Technology, supported by the Defense Advanced Research Project Agency (DARPA), with a mission to develop adaptive optical circuits by integrating optical and fluidic devices [9]. The optics-centric definition of optofluidics revealed the original idea of this field—optics researchers were trying to incorporate microfluidic technologies to create novel optical devices, *i.e.*, the combination of the advantages of optics and microfluidics. After the pioneering paper by Psaltis *et al.* [10] and a review by Monat *et al.* [11], optofluidics is broadly defined as the combination of optics and microfluidics in the same platform to harvest the unique advantages of technologies in these two fields.

For the development of opto-microfluidics, the techniques to fabricate the devices and the applications of the unique capabilities enabled by these devices are the most prominent topics. In this paper, various microfabrication techniques and applications reported by the researchers in Canada will be reviewed. In the following sections, we will first discuss the techniques for microfabrication of opto-microfluidic sensors and then the applications of opto-microfluidic sensors, which include sensors for biological analysis, chemical sensors, surface plasmon resonance (SPR) sensors, and opto-microfluidic sensors integrated with novel optical functionalities. Current research activities in the Photonics Group at the Memorial University of Newfoundland on the femtosecond (fs) laser microfabrication of opto-microfluidic devices are introduced and some experimental results are discussed. Table 1 lists some Canadian research institutions working on microfluidics and optofluidics and their research topics.

The research on microfluidics and optofluidics in Canada involves many research groups in universities across Canada, government agency such as National Research Council of Canada, and companies such as Axela Inc. and Micralyne Inc. These research activities are actively pursued by researchers from different disciplines including chemistry, physics, biomedical engineering, electrical engineering, and mechanical engineering, which reflect the interdisciplinary nature of this diverse research field. We should point out that the review here is intended to give a snapshot of research activities in this field in Canada. However, it is by no mean to be comprehensive due to the scale and rapid progress that this research field evolves. The discussion in this review focuses on opto-microfluidic research in Canada that involves optical elements or optical functionalities but not the purely microfluidic research.

## 2. Techniques for Microfabrication of Opto-Microfluidic Sensors

### 2.1. Molding Fabrication of Opto-Microfluidic Sensors

**Figure 1.**
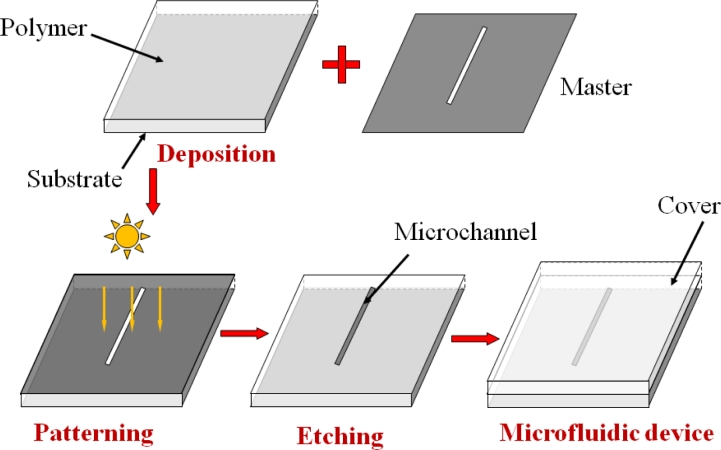
Procedures for the fabrication of a microfluidic device by MEMS technology.

The original microfluidic fabrication technologies were derived from silicon microelectronics, which were well developed in semiconductor industry. However, these techniques are very expensive, complicated, and time consuming. In addition, silicon is not suitable to be applied in a microfluidic device due to its opacification to the visible and UV light in addition to its high cost. Feasible techniques for the fabrication of microfluidic devices include microelectromechanical systems (MEMS) with its procedures illustrated in Figure 1. During the fabrication, a material, especially a polymer, is deposited on a substrate first, and then a pattern in a master is transferred into the material by lithography. After an etching process (wet or dry etching), either the exposed or unexposed material is removed. Finally a cover is attached on the surface of the chip to enclose the microchannels.

**Figure 2.**
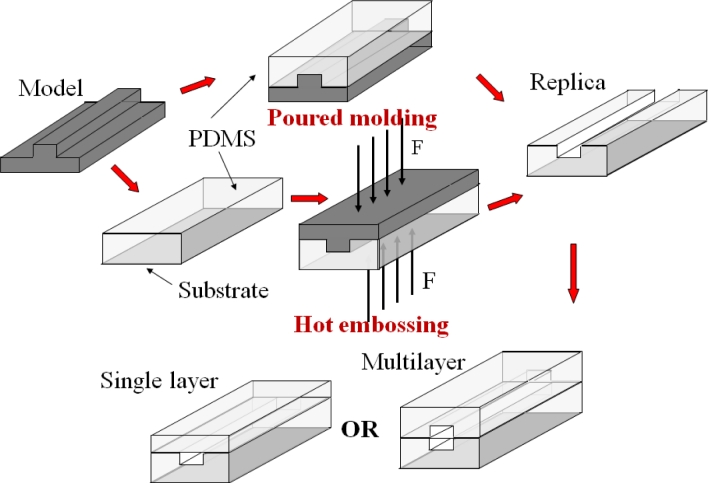
Steps for the fabrication of a microfluidic device by casting.

Another widely adopted method to fabricate a microfluidic system is casting, in which a soft polymer elastomer of high optical transparency is used, *i.e.*, poly(dimethylsiloxane) (PDMS) or poly(methyl methacrylate) (PMMA). Figure 2 shows the steps for the fabrication of a microfluidic device by casting. A mold is produced by soft lithography [12,13] or laser fabrication [14–16] in a photoresist layer (SU-8) or a metallic sheet. Hot embossing technique [14,16,17] or poured molding method [15,18–20] is used to duplicate the mold in a polymer sheet. The peeled polymer replica is sealed to a flat surface to enclose the channels. Much complex structures in a microfluidic device can be developed by stacking multiple polymer layers (100 μm in thickness per layer), similar to a sandwich structure [18,21]. The time period is less than two days starting from design to the realization of a functional device. A list of materials used in the fabrication of opto-microfluidic devices with different molding fabrication techniques is presented in Table 2, which shows that PDMS is the most popular material used in poured molding method and PMMA is widely used in hot embossing technique. In some cases, optical components, especially optofluidic waveguides, have been integrated into the chips and their effectiveness in the transmission of light has been demonstrated. Examples include solid core/solid cladding waveguides [22,23], solid core/liquid cladding waveguides, liquid core/solid cladding waveguides [24], liquid core/liquid cladding waveguides, and hybrid waveguides [18,25,26].

### 2.2. Femtosecond Laser Fabrication of Opto-Microfluidic Sensors

**Figure 3.**
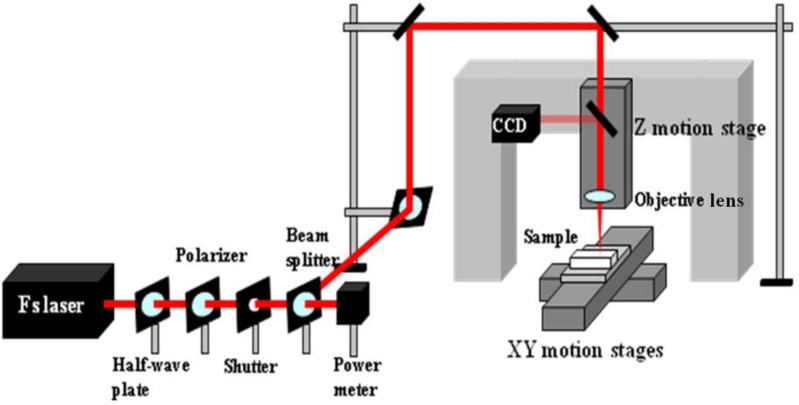
Schematic illustration of an ultrafast laser microfabrication station.

**Figure 4.**
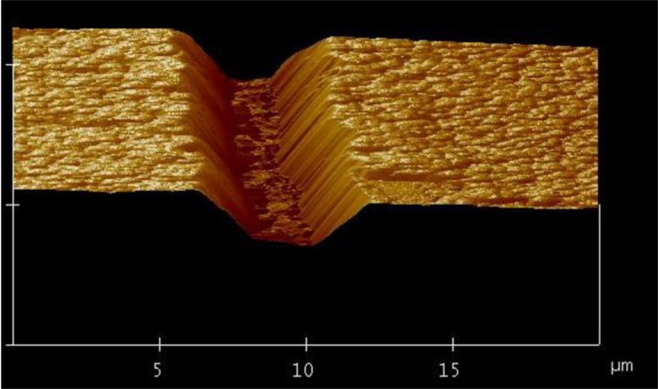
A femtosecond-laser-microfabricated microchannel in fused silica observed by atomic force microscope (AFM).

For the applications in optofluidics, the drawbacks of using polymer materials such as solubility in many common solvents, damage upon tightly focused laser irradiation, and fluorescence at certain common wavelengths appear to be severe. In contrast, the glass-based devices with high optical transparency to visible light and inertia to chemical solvents are very suitable for these applications. Consequently, new fabrication techniques are required to realize microfabrication in various glasses. In this regard, ultrafast laser microfabrication, which utilizes ultrashort laser pulses of pulse width as narrow as ∼100 femtoseconds (a femtosecond laser), has been revealed as a powerful approach to fabricate opto-microfluidic devices in glasses [27]. Figure 3 shows a schematic illustration of a home-made femtosecond laser microfabrication station established in our laboratory at the Memorial University of Newfoundland [28]. By shining a piece of glass with a focused femtosecond laser of a peak intensity well above the damage threshold of the glass, three-dimensional microstructures could be generated in the glass. Figure 4 shows a femtosecond-laser-microfabricated microchannel in a fused silica observed by an atomic force microscope (AFM), indicating widths at the top and bottom of the channel of ∼5 and 2 μm, respectively.

**Figure 5.**
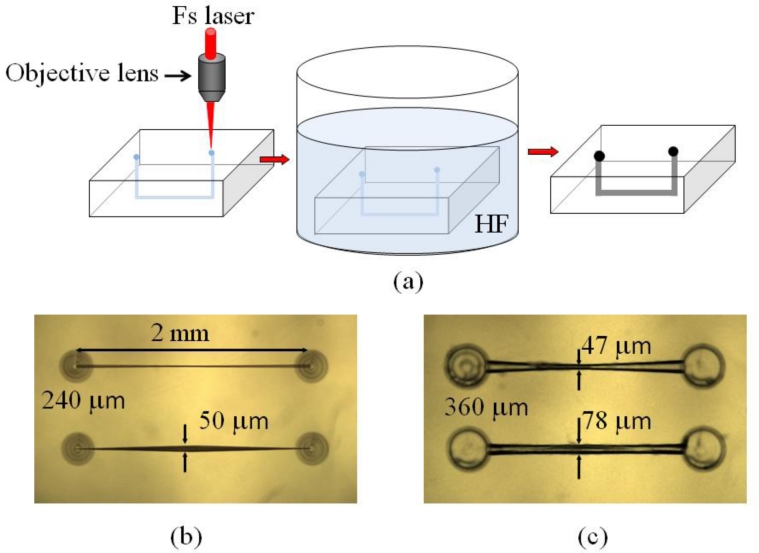
Femtosecond laser microfabrication of opto-microfluidic devices: (**a**) fabrication procedures, (**b**) U-shape-microchannels in fused silica before etching, and (**c**) after etching for 5 hours in 20% HF acid within a shaker.

After hydrofluoric (HF) acid etching for 3–5 hours, microchannels are generated, which can be adopted in opto-microfluidic devices. The procedures for the fabrication of opto-microfluidic devices with a femtosecond laser are shown in Figure 5(a). Examples of opto-microfluidic channels and microstructures fabricated in our group are shown in Figure 5(b,c).

**Figure 6.**
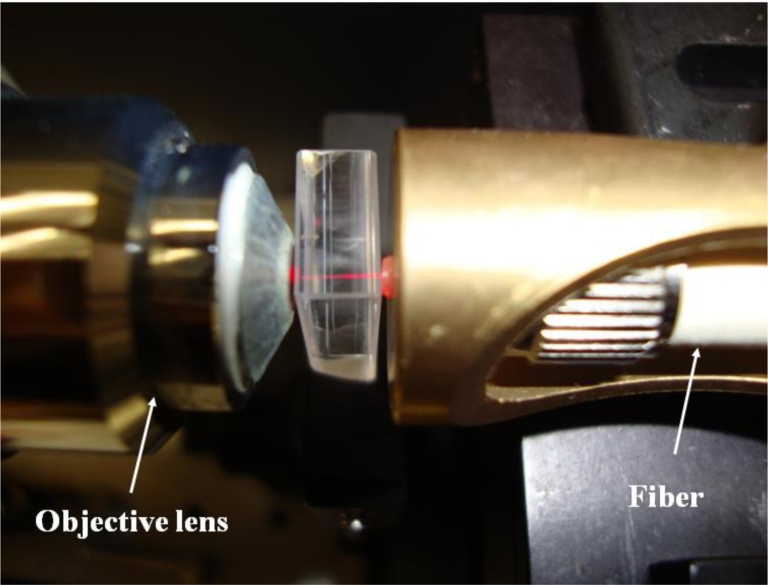
Type II waveguide coupling in fused silica using a He-Ne laser at 633 nm.

Compared with MEMS techniques, the ultrafast laser microfabrication possesses salient advantages of simplicity, high efficiency, and versatility. In addition to the fabrication of microchannels, optical waveguides can also be fabricated by an ultrafast laser [29–31]. When a femtosecond laser beam with pulse energy of several hundred nano-Joules is irradiated on the glass, the refractive index of the region in glass will increase. This type of waveguide is called a Type I waveguide. Another type of waveguide, *i.e.*, Type II waveguide, can be fabricated with higher peak energy. Laser irradiation with peak intensity higher than the damage threshold of the glass causes the material at the focus to vaporize, thus the high pressure pushes the material to both sides of the irradiated region. Consequently, the tracks at the two sides result in a higher refractive index while the region between the tracks guides light. In this case, the distance between the two tracks should be shorter than 50 μm. Figure 6 is a Type II waveguide coupling with a He-Ne laser at 633 nm. The propagation loss of this kind of waveguide is ∼4 dB/cm, which is somewhat higher than the values of waveguides fabricated by other techniques. However, the propagation loss in an optical waveguide fabricated by a femtosecond laser could be reduced after optimization of the fabrication parameters including the laser properties. The relatively higher propagation loss does not have a serious effect for the adoption of these waveguides in opto-microfluidic devices.

Table 3 lists a comparison of the properties of some reported optofluidic waveguides fabricated by different techniques. Among three leading fabrication technologies, *i.e.*, MEMS, casting, and femtosecond laser microfabrication, the resultant optofluidic waveguides have quite different light propagation properties. It is important to keep in mind that the propagation loss of a waveguide is relative to the material property of the core and cladding (refractive index) and the carried wavelength. For the waveguides fabricated by MEMS or casting techniques, their core and cladding materials can be either liquid or solid. The reported propagation losses of the waveguides fabricated by MEMS techniques range from 0.5 to 4 dB/cm while the values for the waveguides fabricated by molding fall in a range of 1.8–8.2 dB/cm. The femtosecond laser microfabrication technique is a powerful method to fabricate opto-microfluidic waveguides, in particularly on glasses. With the Type I or II waveguides fabricated with different peak intensities of the lasers, different propagation losses of the waveguides have been achieved, in which values around ∼1 dB/cm were found for the Type I waveguides and slightly higher propagation losses for the Type II waveguides.

Any of the aforementioned microfabrication techniques is effective to fabricate opto-microfluidic devices with pros and cons. Table 4 compares the advantages and drawbacks of different microfabrication techniques used for fabricating opto-microfluidic sensors. Judicious selection is necessary in order to choose a suitable technique for a specific application. In some cases, special approach may be required to overcome the drawbacks of specific microfabrication technique.

## 3. Applications of Opto-Microfluidic Sensors

### 3.1. Opto-Microfluidic Sensors for Biological Analysis

The original motivation for developing opto-microfluidics was for biological analysis, in which immunoassay and DNA separation have been the two main applications. Immunoassay is a biochemical technique that detects the presence and quantities of antibodies or antigens in the samples [32–34]. Competitive immunoassay and sandwich assay are two common methods [28]. When a labeled (such as an enzyme or fluorescent dye) analytical reagent with special antigens or antibodies is mixed with the sample, a specific binding will form between the antigen and the corresponding antibody. The changes in the color and intensity of light from the labeled binding are recorded to identify the immunity of the sample. The traditional technique requires a large amount of reagents and a long incubation time. Microfluidics-based immunoassays have been developed by several research groups. For instance, in a sandwich assay for antigen, antibody is injected into the microchannels by pressure-driven [35], electrokinetic control [36,37] or centrifugal force [38], and deposited onto the wall of the microchannels to form solid-phase antibody, then antigen in the sample is mixed with solid-phase antibody in the microchannel, and finally another labeled antibody is added for signal generation. The color signals from the antibody-antigen-labeled antibody bindings are detected by a fluorescence microscope after removing excess antibodies and antigens through washing steps. All steps can be completed in one hour, and the reagent consumption is only on the order of microliters. Roos *et al.* [39] and Herrmann *et al.* [40] used microspheres to support the binding, and the fluorescence signals were collected from these microbeads. These microspheres provided increased surface area to support the immune complex. Nanoparticle-labeled microfluidic immunoassay [41] was reported by Lin *et al.*, in which free labeled antigens were deposited on the nanoscaled gold particles and the scattered light from the particles provided readout to trace the bindings between antibodies and antigens.

As a prerequisite step to allow opto-microfluidic measurement, capillary electrophoresis (CE) is a rapidly growing separation technique, which has been applied in bioanalysis, environmental pollutant analysis and food analysis [42,43]. By using high electric fields (larger than 500 V/cm), buffer solution and all ions, positive or negative particles in the sample generate electroosmotic and electrophoretic flow respectively. Electroosmotic flow pulls the analytes in the buffer solution through the capillary toward the cathode. However, the electrophoretic flow reduces the flow rate of the negatively charged analytes and increases the flow rate of positively charged analytes in the capillary. Therefore, the analytes separate due to different mobilities. Ultraviolet (UV) absorption detectors are equipped to detect the ingredients of the sample near the outlet of the capillary. The data from the detector is displayed as an electropherogram, in which peaks at different times are shown for separated chemical compounds.

**Figure 7.**
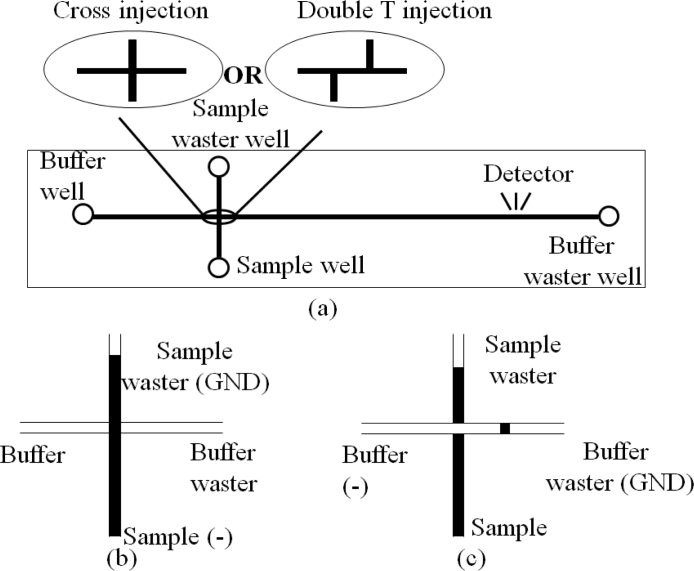
Schematic illustration of a CE chip: (**a**) a CE chip with reservoirs, (**b**) CE process of the injection phase, and (**c**) CE process of the separation phase.

A CE instrument is formed after assembling the microchannels and reservoirs in a chip with extra high potential. Figure 7 illustrates two types of CE chips and their operations. The CE chips are extensively used in medical research such as DNA analysis [44], infectious disease diagnostics [45] and sample purification [46]. Now some companies such as Micronit Microfluidics BV in The Netherlands, microLIQUID in USA, and Micralyne Inc. in Canada supply CE chips and kits for clinical diagnostics. Meanwhile, several research groups have integrated different components into the CE chips to satisfy diverse needs of analysis. For example, combination of polymerase chain reaction (PCR) with CE chip (PCR-CE chip) is a focus of research, in which one or multiple PCR chambers are integrated into the CE chip to incubate and analyze DNA simultaneously [47–52]. Prakash *et al.* fabricated PCR arrays on the CE chip with two PCR valves and ports to control the flow of samples in each PCR chamber, so that the CE chip can be reusable without contamination [53]. CE chips integrated with multichannels for mixing, reaction and separation were also reported by some Canadian research groups [54–57]. Munce *et al.* used CE with optical tweezers to achieve single-cell sorting and analysis in one chip [58]. A CE chip with an acoustic wave sensor successfully trapped one live myocyte in the acoustic wave sensor region [59], in which the contraction and relaxation of the cell were clearly detected without the influence from other cells.

**Figure 8.**
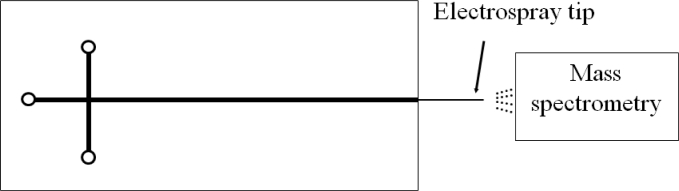
Schematic illustration of a CE chip with electrospray mass spectrometry.

The commonly adopted detection method in a CE chip is a confocal detective system. Bliss *et al.* integrated optical waveguides into a CE chip [60]. However, the electropherogram demonstrated that the waveguide detection possesses an equal or greater sensitivity than that of the confocal system. A good technique to accurately identify the ingredient of a sample is to connect the outlet of the capillary to an electrospray mass spectrometry (ESMS) [52], as shown in Figure 8.

### 3.2. Opto-Microfluidic Chemical Sensors

Polymer particles are highly sought in biology, cosmetics, food process, medicine, and pesticide. However, the incomplete control and high cost of traditional chemical synthesis techniques limit their applications. One research group at the University of Toronto proposed a novel approach to realize continuous and scalable production of core-shell droplets and polymer capsules in microfluidic devices in 2005 [61]. Two or more different kinds of immiscible liquids were infused into a microfluidic device and the mixed liquid flew through a thin “focused” orifice. Different core-shell droplets were synthesized by changing the liquid flow rates, which were controlled by push pumps. UV lamp irradiation made the photopolymerizable droplets solidification when droplets flew along a long wavy microchannel. Polymer particles were then obtained at the output of the device. This technique could precisely control the sizes of the particles, liquid cores, shells, and the number of core droplets. During the subsequent years, further research had been carried out by the same group to address additional topics, such as biopolymer microcapsules [62], Janus and ternary particles [63], microgels of biological polymers [64], monodisperse particles with microspheres, rods, disks and ellipsoids [65], dynamic lattices [66,67], and colloidal particles at gas–liquid interfaces [68]. This kind of chemical sensors could better explore the dynamics and mechanisms of physical and chemical reactions which are difficult to be investigated in conventional experiments.

Centrifugal microfluidic devices were reported by a research group at McGill University, in which multiple chambers and capillary valves were fabricated in a disc. The flow of the solvent was controlled to achieve dissolution [69], mixing [70], extraction [21,71] and reaction [70] between reagents by adjusting the rotational rate of the disc. The components and concentrations of analytes were measured at the detection unit by fluorescence and absorption spectra. In some cases, magnets were utilized in the chamber and base to serve as an agitator to increase the efficiency of chemical reaction. The centrifugal microfluidic devices could be applied in environmental monitoring and soil analysis for their portability and rapidity.

Optofluidic platform for chemical component analysis by laser-induced breakdown spectroscopy (LIBS) was achieved by a research group at the University of Alberta [72,73]. A small thermal or piezoelectric actuator was deposited on the bottom of a microchannel while an orifice of a few microns was opened on the opposite wall of the actuator. During the operation, a bubble was first excited at the orifice by the actuator, and then a fast intense laser pulse broke down the bubble, and thus plasma with the components of reagent was generated. As the plasma was cooled down, electrons and ions were recombined, accompanied by emitting electromagnetic radiation with fingerprint wavelengths of the elements in the reagent, which was detected by its spectroscopy. The most notable advantage of this technique is the non-contact measurement, which completely avoids sample contamination.

### 3.3. Opto-Microfluidic Surface Plasmon Resonance Sensors

**Figure 9.**
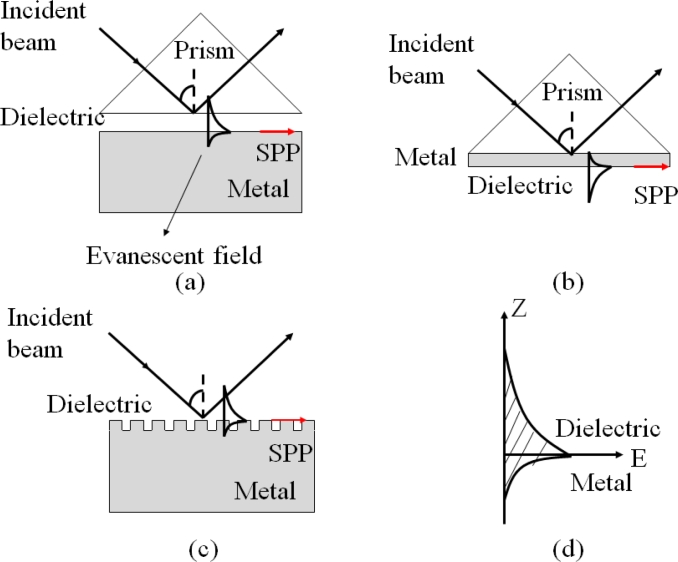
Surface plasmon resonance: (**a**) Otto configuration, (**b**) Kretschmann configuration, (**c**) a diffraction grating, and (**d**) evanescent field at the interface.

Surface plasmon polaritons (SPP) are electromagnetic waves that propagate along a metal and dielectric interface [74,75]. SPP can be evanescent, which are excited by both electrons and photons. The excitation of SPP by photons is usually referred as surface plasmon resonance (SPR). SPP cannot be directly excited by photons due to momentum mismatch. Special arrangements, such as Otto configuration [76], Kretschmann configuration [77], or a diffraction grating [78], are well-known techniques to couple photons into SPP in order to match the wave vectors of the photon and the surface plasmon, as illustrated in Figure 9. The Kretschmann configuration is the most common approach, in which the thickness of the metal layer is usually a few tens of nanometers to ensure the evanescent wave to travel through the metal and couple to a surface plasmon mode, which is shown in Figure 9(d).

Since the wave travels on the interface of the metal and the external dielectric layer, SPR is very sensitive to any change at this interface, such as the density and temperature of the dielectric layer and the structure of the metal surface. Opto-microfluidic SPR sensors for immunoassay and refractive index measurement have been demonstrated. Chien *et al.* integrated SPR into a microfluidic platform [79], in which diffractive mirrors were attached on the prism to focus the incident light in the direction of wave vectors while liquid solution as the dielectric material was infused into a gold-coated microchannel. When light with different incident angles is irradiated on the surface of the prism and metal, part of the light, which matches with the SPR angle *θ_SP_*, is attenuated into the metal to generate SPR and the rest of the light is reflected. A photodetector can be placed at the end of the device to capture and monitor the intensity of the reflected wave. The reflected light of reduced intensity at the SPR angle would be detected due to the optical absorption by the metal. The sensor has been proved to be effective in measuring refractive indices of liquids by monitoring changes in the SPR angles with NaCl solution as a testing material.

Nanohole based SPR sensors have received considerable attention in recent years. One of the most important characteristics of a nanohole array is that the intensities of the transmitted light enhance at certain wavelengths due to the SPP coupling when the incident light irradiates on the surface of the metal film. By monitoring the wavelengths of the intensity peaks, a nanohole-based SPR sensor is realized. Compared with a standard reflective mode SPR sensor, a nanohole SPR sensor operates at the normal incidence without the necessity to consider the SPP angle. Sinton group at the University of Victoria realized nanohole array SPR sensors [80–85]. For example, a device with flow-through nanohole arrays, instead of dead-ended nanohole arrays in a SPR sensor, could make the regents to travel through the nanoholes rapidly, thus reducing the response time remarkably [85].

With the improvement of SPR techniques, surface plasmon resonance imaging (SPRI) has been proposed as a new kind of detection technique in biology [86]. Kanda *et al.* at the University of Alberta patterned a surface of metal (gold) with free-labeled protein antigens arrays [87]. When the sample flew through the microchannel located above the patterned gold film, the antigen-antibody bindings were generated at the arrays. A high contrast SPRI based on the adsorbed proteins was realized to evaluate the quantitative and qualitative properties of the antibodies in the sample. Tabrizian group at the McGill University replaced the plain gold film in the SPR with periodic gold nanoposts to detect DNA hybridization. The optimal result showed a fivefold SPRI enhancement compared with the common SPRI [88]. This group also combined SPRI technique with digital microfluidics (DMF) to detect biological samples [89,90]. DMF is a fluid manipulation technique, in which a patterned array of electrodes is etched on the substrate with MEMS techniques and then coated with a waterproof material like Teflon to form the DMF. Droplets can dispense and merge in the DMF when an electrical potential is applied on sequential electrodes in the array [91–95]. Therefore, this combination realized real-time monitoring and detection of reaction occurring as required.

### 3.4. Opto-Microfluidic Sensors Integrated with Novel Optical Functionalities

Integrating micro-optical components into a chip could effectively shape beams to enhance the sensitivity of a sensor and increase its portability. Besides optical waveguides discussed in the previous sections, Table 5 lists several micro-optical components integrated in opto-microfluidic devices, which include lenses [96,97], gratings [22,98], mirrors [79,99], and light sources [100,101]. There is continuing effort to realize integration of multiple components to acquire novel optical functionalities.

Optical tweezers are important instruments to trap and sort particles like cells, proteins, and microspheres. A tightly-focused laser beam (Gaussian beam) provides an attractive or repulsive force on the particles due to the changes in the momentum of light upon reflection or refraction. Although the force is on the order of piconewtons, it is large enough to hold and move small objects with sizes of several tens of microns. Taking advantages of the merits of opto-microfluidic devices with narrow channel widths, fiber-microfluidic tweezers have been particularly attractive in studying a variety of biological systems. Sinton group realized trapping microsphere array by dual-beam with a focus on the study of optohydrodynamics and optical interactions between particles, which could be applied for contact-free storage of biological cells as well [102–105]. In addition, Guo *et al.* at the University of Ottawa fabricated a microfluidic chip with on-chip lens structures to reduce the beam waist of the light and achieved a higher efficiency in optical operation and optofluidic transportation [96].

Microcytometer is another important application of opto-microfluidic sensors. Azmayesh-Fard *et al.* [18] coupled excitation lasers of different wavelengths into the microfluidic devices by solid-core waveguides and different dyed particles could be resolved from different peaks after a windowed Fourier transform to output signals. Multiple parallel waveguides were integrated into an opto-microfluidic sensor to collect the fluorescent and scattered signals from the labeled cells by Xu group in McMaster University [106]. The same group also fabricated micro-lenses in the chips to shape the excitation beam and improve the signal-to-noise ratio of cytometers [97]. A wide-angle microfluidic cytometer was reported by a research group at the University of Alberta [107–109]. A conventional cytometer can only collect signals through small-angle forward scattering (5°) and side scattering (10°) in which errors are apparent when cells gather or irregular cells and other organelles exist. In the wide-angle microfluidic cytometer, the goniometric measurement and finite-difference time-domain (FDTD) method were adopted to overcome the disadvantages of the conventional cytometers.

Refractive index (RI) sensing is another important application of opto-microfluidic sensors, which have been widely applied in environmental monitoring and optical measurement. Opto-microfluidic RI sensors based on Mach-Zehnder interferometer (MZI) [23,110,111], grating [98,112], and refractometer [99] have been reported by several research groups in Canada. All these reported methods adopted similar principles in which two identical beams were first coupled into different paths such as a solid core waveguide or a liquid core waveguide, then interference occurred due to the difference in the optical paths when the two beams were combined together. The measurement of the refractive index of the liquid can be achieved by monitoring the change of the interference intensity.

## 4. Conclusions

Research on opto-microfluidics has achieved significant progress over recent years. Considering some successful microfluidic devices and systems, optofluidics is a field still in its infancy with plenty room for further development and promising applications. With the development of advanced microfabrication techniques, more advanced opto-microfluidic sensors with increased functionalities and compactness will be developed. One important aspect for further investigation is on the integration of microfluidic platforms and fluid actuation techniques to allow efficient interactions with optical signals. One the other hand, optical components and mechanisms are crucial to achieve optical control of fluids. An enhanced understanding of the interplay between optics and fluids will make it possible to realize reconfigurable optical systems with novel functionalities. Discoveries in other research fields will benefit the development of opto-microfluidics. For example, smaller waveguide (nano-waveguide or sub-wavelength waveguide) or nanofluidics will be interesting for realizing nanodevices and nanosystems with higher integration density. Significant achievement is anticipated through extensive collaborations among researchers from different disciplines as well as from different countries and regions to address topics in this interdisciplinary field.

## Figures and Tables

**Table 1.** List of some Canadian research institutions working on microfluidics and optofluidics.

**Institution**	**Group**	**Topic**	**Website**
Concordia University	Optical-Bio Microsystems Laboratory	Lab-on-a-chip, microfluidics, micromachining	http://users.encs.concordia.ca/~mpackir/
École Polytechnique de Montreal	NanoRobotics Laboratory	Micro/nanofabrication, lab-on-chip, biomedical	http://wiki.polymtl.ca/nano/index.php/NanoRobotics_Laboratory
McGill University	David Juncker Nanobioengineering Group	Microfluidic probe, biomedical	http://wikisites.mcgill.ca/djgroup/index.php/Main_Page
McGill University	Salin Group	Disc-shape centrifugal microfluidics, environment analysis	http://salin-group.mcgill.ca/lab.html
McGill University	Tabrazian Group	Biomedical, plasmonic biosensors	http://www.mcgill.ca/dentistry/research/tabrizian/
McMaster University	Centre for Advanced Micro-Electro-Fluidics	Microfluidics, lab-on-chip, bioMEMS, biomedical	http://mech.mcmaster.ca/camef/index.html
McMaster University	Xu Group	Microfluidic flow cytometer	http://engphys.mcmaster.ca/faculty_staff/faculty/xu/
Memorial University of Newfoundland	Photonics Group	Femtosecond laser microfabrication, opto-microfluidics, nanophotonics, fiber sensing	http://www.mun.ca/physics/people/faculty/chen.php
National Research Council of Canada	Laser and Materials Processing Group	Laser microfabrication, microfluidics	http://www.nrc-cnrc.gc.ca/eng/facilities/imi/camm.html
Queen’s University	Oleschuk group	Nanoelectrospry ionization mass spectrometry, microfabrication	http://www.chem.queensu.ca/people/faculty/oleschuk/index.html
Simon Fraser University	Microinstrumentation Laboratory	Biomedical, interconnect for microfluidics, microneedle array	http://mil.ensc.sfu.ca/
University of Alberta	Tsui Group	Opto-microfluidic cytometer	http://www.ece.ualberta.ca/~tsui/
University of Alberta	Micro & Nano-Scale Transport Laboratory	Lab-on-chip, biomedical, energy application	http://www.mece.ualberta.ca/mntl/
University of Alberta	Harrison Group	Proteomics, multiplexed systems	http://www.chem.ualberta.ca/~harrison/index.html
University of British Columbia	Hansen Group	Microfabrication, microfluidics, lab-on-chip, biomedical	http://www.chibi.ubc.ca/faculty/hansen/labhome
University of British Columbia	Microsystems and Nanotechnology Group	MEMS, biomedical	http://www.mina.ubc.ca/MiNa_about
University of Calgary	Biosystems Research and Applications Group	Digital microfluidics, biomedical	http://www.brag.ucalgary.ca/index.html
University of Manitoba	Immuno trafficking Lab	Microfluidics, lab-on-chip, biomedical	http://www.physics.umanitoba.ca/~flin/index.html
University of Toronto	Aitchison Group	Lab-on-chip	http://photonics.light.utoronto.ca/aitchison/index.html
University of Toronto	Goh Group	Biosensors	http://www.chem.utoronto.ca/staff/MCG/home.html
University of Toronto	Herman Group	Femtosecond laser microfabrication, microfluidics, lab-on-a-chip	http://photonics.light.utoronto.ca/laserphotonics/
University of Toronto	Kumacheva Microfluidics Group	Microfluidic synthesis of particles, biological environments, biomedical	http://www.chem.utoronto.ca/staff/EK/index.htm
University of Toronto	Wheeler Lab-on-a-Chip Group	Digital microfluidics, biomedical	http://www.chem.utoronto.ca/staff/WHEELER/html/Main.htm
University of Toronto Mississauga	Krull Group	Biomedical, plasmonic biosensors	http://www.utm.utoronto.ca/index.php?id=10138
University of Victoria	Gordon Group	Microfluidics, surface Plasmon resonance, nanophotonics	http://www.ece.uvic.ca/~rgordon/research.html
University of Victoria	Sinton Group	Plasmonic biosensors, micro- and nanofluidics, micro fuel cells, optical tweezer	http://www.microfluidics.uvic.ca/
University of Waterloo	Advanced Micro-/Nano-Devices Lab	MEMS/NEMS, microassembly, biomedical	http://biomems.uwaterloo.ca/index.html
University of Waterloo	Mechanical & Mechatronics Engineering	MEMS, biomedical, microfluidic	http://www.mme.uwaterloo.ca/people/group.php

**Table 2.** Materials used in opto-microfluidic devices by different molding fabrication techniques.

**No.**	**Material**	**Fabrication Technique**	**Ref.**

1	Photoresist (SU-8)	MEMS	[20,26]
2	Ge-doped silica	MEMS	[23]
3	Glassy carbon (GC)	MEMS	[13]
4	PDMS	Poured molding	[12,14,15,18–20,24]
5	PMMA	Hot embossing	[12,14,16]
6	Poly(cyclic olefin) (PCO)	Hot embossing	[17]

**Table 3.** Comparison of the properties of optofluidic waveguides fabricated by different techniques.

**No.**	**Ref.**	**Method**	**Core**	**Cladding**	**Propagation loss**
1	[22]	MEMS	Silicon	SU-8 and silica	2.1 dB/cm at 1,550 nm
2	[23]	MEMS	Oil	Oxidized silicon and borophosilicate glass	0.5 dB/cm at 1,500 nm
3	[23]	MEMS	Ge-doped silica	Oxidized silicon and borophosilicate glass	0.94 dB/cm at 1,500 nm
4	[18]	Casting	PDMS	PDMS and air	3.1 dB/cm at 532 nm, 2.9 dB/cm at 633 nm
5	[24]	Casting	Glycerol	Sudan-doped PDMS	8.2 dB/cm at 532 nm, 1.1 dB/cm at 633 nm
6	[25]	Casting	Silicon	H_2_O and silica	3 – 4 dB/cm at 1,550 nm
7	[14]	Casting	Liquid PDMS	PDMS	1.8 dB/cm at 532 nm, 1.0 dB/cm at 633 nm
8	[29]	fs laser (type I)	Fused silica		0.3–0.4 dB/cm at 1,550 nm
9	[30]	fs laser (type I)	Fused silica		1 dB/cm at 1,550 nm
10	[31]	fs laser (type I)	Crystalline silicon		1.2 dB/cm at 1,320 nm, 0.7 dB/cm at 1,550 nm
11	*	fs laser (type II)	Fused silica		4 dB/cm at 1,550 nm

* Data obtained from the Photonics Group, Memorial University of Newfoundland.

**Table 4.** Comparison of the advantages and drawbacks of different fabrication techniques.

**Fabrication technique**		**Advantages**	**Drawbacks**	**Ref.**
Molding fabrication	MEMS	Possibility to fabricate complex structures	Long fabrication time; Fluorescence of polymer at certain common wavelengths; Material damage upon tightly focused laser irradiation; Polymer solubility in many common solvents.	[12,15–17,23]
	Casting			
Femtosecond laser fabrication		Less fabrication time Optical transparency to visible light Inertia to chemical solvent	Requiring precise laser focus and motion control; Possible requirement of additional chemical etching.	[27,29,30]

**Table 5.** Micro-optical components integrated in opto-microfluidic devices.

**Optical component**	**Fabrication technique**	**Ref.**
Lenses	MEMS	[96,97]
Gratings	MEMS Femtosecond laser	[22,98]
Mirrors	MEMS	[79,99]
Light sources	MEMS Femtosecond laser	[100] (USA) [101] (Italy)
